# Endoscopically assisted transcutaneous placement of a balloon catheter in the medial guttural pouch compartment of the horse: A surgical approach to local treatment

**DOI:** 10.1111/vsu.70059

**Published:** 2025-11-20

**Authors:** Héloïse Lepage, Charles de Chaisemartin, Antonella Spadaro Rosselo, Hélène Leroy, Olivier Lepage

**Affiliations:** ^1^ Department of Morphology and Pathology, Faculty of Veterinary Medicine University of Liège Liège Belgium; ^2^ Center for Equine Health, Ecole Nationale Vétérinaire de Lyon, VetAgro Sup University of Lyon Marcy l'Etoile France

## Abstract

**Objective:**

To describe and report clinical outcomes after transcutaneous guttural pouch (GP) catheterization (TGPC) in standing horses.

**Study design:**

Ex vivo study and case series.

**Animals:**

One cadaver head, records of 10 normal horses and 14 horses treated with TGPC.

**Methods:**

Relevant anatomical landmarks were determined through dissection of one cadaveric specimen and 10 normal radiographic studies. Records of horses diagnosed with empyema or mycosis and treated with standing TGPC were reviewed for complications that occurred during or after the operation.

**Results:**

Ex vivo and radiological studies identified the tissues crossed by the catheter and anatomical variations of the stylohyoid. TGPC performed on one (*n* = 13) or both (*n* = 1) GPs was successful in all cases. The balloon catheter placed in the parotid region allowed administration of oxygen and lavage solution and facilitated passive or active drainage. Complications included hemorrhage from the skin (3/15; 21%), catheter balloon rupture (1/15; 7%), cutaneous salivary fistula (1/15; 7%), abrasions under the fixation ring (15/15; 100%) and catheter dislodgement (3 of 500 treatment sessions). The balloon catheter remained in place for 4 to 17 days. Following catheter removal, sealing of the GP was achieved within 72 h; second‐intention healing was complete in less than 10 days.

**Conclusion:**

TGPC in standing horses was frequently associated with minor complications but allowed local treatment for up to 17 days.

**Clinical significance:**

This study provides evidence to support the transcutaneous placement of a 20 Fr balloon catheter in the GP for local treatment.

## 1 INTRODUCTION

In horses, treatment of the guttural pouch (GP) presents a challenge,^1^ given the anatomy and difficult access. Both GPs are located caudal to the rami of the mandibles, dorsocaudal to the nasopharynx and larynx, and ventral to the base of the skull and wing of the atlas. They lie deep to the parotid salivary glands and have anatomical relations with adjacent structures, including several cranial nerves, maxillary vein and artery, internal carotid artery (ICA), and external carotid artery (ECA). Knowledge of the GP endoscopic anatomy is essential for accurate diagnosis and subsequent treatment.^2^

In cases of GP mycosis or empyema, it is often necessary to administer repeated local treatment,^3^, ^4^ and to provide appropriate drainage.^4^, ^5^ This can be achieved using a transnasal approach with a GP lavage catheter^4^ or directly using an endoscope lavage catheter.^6^ In some cases, surgical access is required to remove purulent debris that may become inspissated over time.^7^ The surgical method used to gain direct access to the lateral compartment of the GP is the modified Garm approach.^8^ Access to the medial compartment is achieved using a hyovertebrotomy, a Viborg's triangle, a Whitehouse, or a modified Whitehouse approaches.^9^ A 28 Fr Foley catheter inserted through the cutaneous incision of a modified Whitehouse approach has been described to assist in lavaging the GP.^7^ However, all these surgical methods carry a risk of damaging large blood vessels or cranial nerves when penetrating the wall of the GP. If the GP is infected with fungus or contains inspissated exudate, the swelling can vary, which makes surgery more difficult due to the depth of dissection required.^7^

Endoscopic salpingopharyngostomy with a diode laser in sedated horses is advocated to hasten resolution of chronic GP disease,^10^ tympany with secondary GP empyema^11^ while also modifying the GP environment to treat mycosis. This approach avoids the need for general anesthesia, the risk of iatrogenic nerve damage, and the postoperative wound management required with an open transcutaneous surgical approach.^10^

To the best of our knowledge, there has not yet been a study of a detailed description of a minimally invasive external approach that can provide repeat treatments for GP empyema and mycosis while avoiding long wound closure times. The objective of this case series was to report the outcomes and associated complications of transcutaneous GP catheterization (TGPC). We hypothesized that TGPC, performed under endoscopic guidance in sedated horses, would allow a 20 Fr balloon catheter to be placed in the medial compartment of the GP. This would facilitate repeated treatments with minimal complications and a short wound closure time.

## 2 MATERIALS AND METHODS

### 2.1 Ex vivo anatomical study

The proposed catheter penetration site (CPS) was identified using the left side cadaver head of a 2.5 year old, 600 kg mare of unknown breed. The CPS is defined as the center of a circle 1 cm in diameter in which a catheter, perpendicular to the skin, is correctly placed to penetrate the medial compartment of the ipsilateral GP.

#### 2.1.1 External body landmarks

**FIGURE 1:**
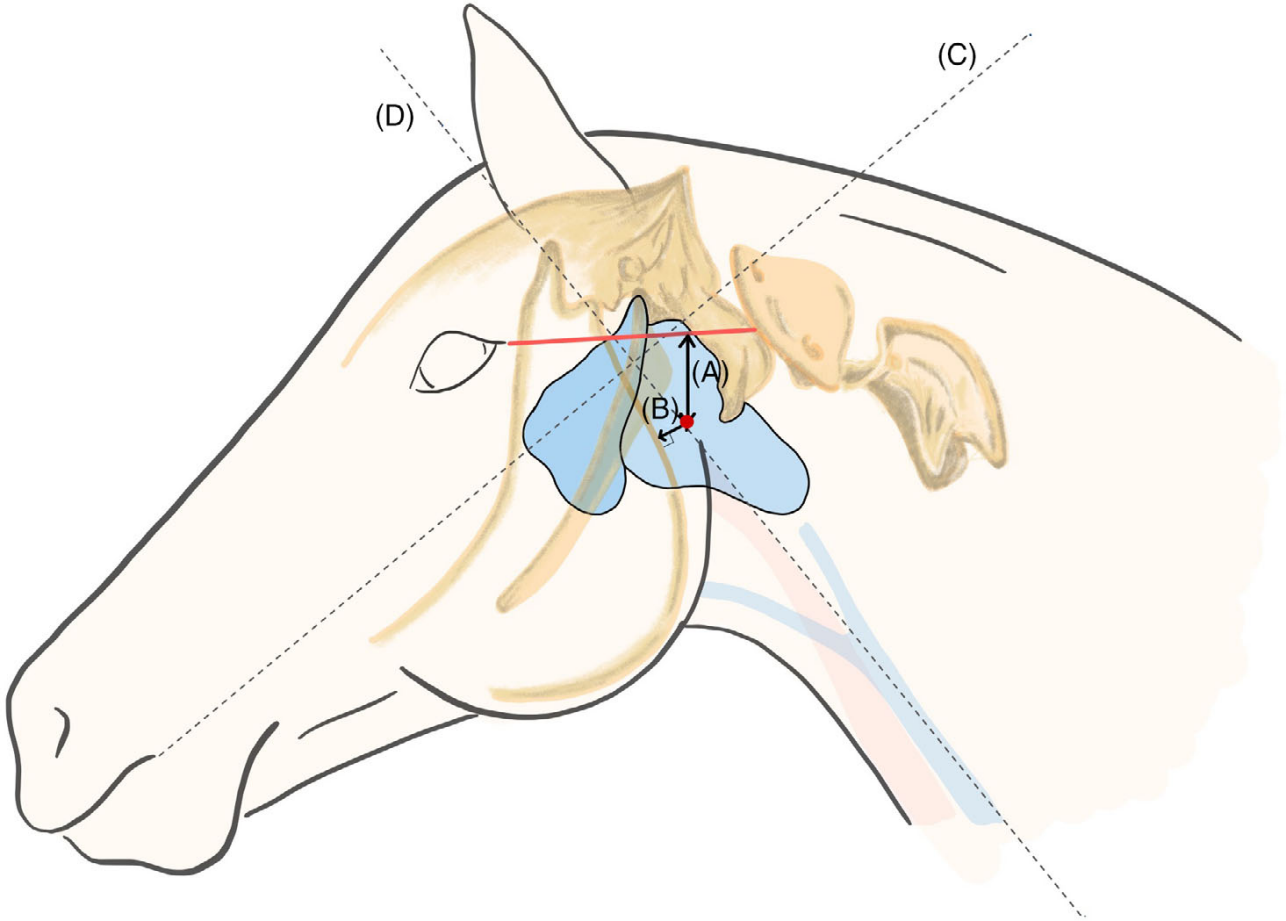
Drawing representing the external body landmarks. The catheter penetration site is located 4 cm (A) ventral to a virtual line connecting the middle of the atlas wing to the lateral canthus of the eye and 3 cm (B) caudal to the vertical ramus of the mandible, with the head in line with the neck and held at a 90° angle. The 90° body angle is established by crossing, in the parotid region, a line parallel to the jugular groove (D) and a line extending from the ipsilateral commissure of the mouth (C).

Head position was determined by two lines which crossed at a 90° angle in the parotid region. The first line was parallel to the jugular groove and the second line extended from the ipsilateral commissure of the mouth. Using these lines, the head was placed in a 90° angle. With the head in this position, the CPS was identified as 4 cm below a line connecting the middle of the wing of the atlas to the lateral canthus of the eye, 3 cm caudal to the vertical ramus of the mandible (Figure 1).

#### 2.1.2 Topographical anatomy

Approximately 500, 1000, and 10 mL of colored latex were injected into the left common carotid artery, jugular vein, and parotid duct, respectively. After allowing the latex to solidify, a 14 gauge 2.1 × 133 mm IV catheter (BD Angiocath, Becton Dickinson, Utah) was inserted at the CPS, and a detailed dissection of the surrounding area was performed.

### 2.2 In vivo radiological study

A skin staple was placed at the CPS in 10 horses without GP pathology, and a lateral radiographic projection of the head at a 90° body angle was obtained from each horse. A consent form authorizing this procedure was obtained from the owner or representative. The relation between the staple placed at the CPS and the stylohyoid bone was assessed.

### 2.3 Study population

Cases of GP mycosis or empyema presented between December 2023 and February 2025 at a single European academic hospital were reviewed. A consent form approved by the Animal Experimentation Ethics Committee (CE‐2495, Comité d'éthique en expérimentation animale, VetAgro Sup, France) authorizing the use of TGPC was obtained from the owner or representative.

### 2.4 Data collection

Signalments and medical histories, including other surgical procedures to treat GP, were obtained for each clinical case (Table 1). Hospitalization data recorded included the side affected; abnormality detected on parotid palpation (swelling, pain, and increased temperature); appearance or worsening, if present on admission, of a cranial nerve deficit; findings on endoscopy during TGPC, every 5 days, and at removal of the balloon catheter. Clinical and surgical findings related to TGPC were also recorded. Any variations to the original procedure described in section 2.5 were defined as complications and reported as either minor or major, the latter requiring surgical revision. The methods used to prevent or treat the complications were also recorded. The owners were questioned over the telephone 10–15 days after the catheter was removed. Information obtained included of the patient status (dead or alive); clinical description of the CPS region (swelling, pain, or increased temperature); and an image of the surgical site 10 days after catheter removal.

**TABLE 1:** Summary of clinical data for 15 transcutaneous guttural pouch catheterization (listed in chronological order) in 14 horses.

P	Signalment	Affected guttural pouch	Presenting disease	Concomitant surgery	Therapy		Complication during the catheterization procedure		Complication during treatment			Duration of catheterization
	Breed, sex, age	L/R	M/E		Oxygen	Lavage	Venous bleeding at skin level	Drops of blood at guttural pouch mucosa level	Inadvertent catheter removal	Balloon rupture	Salivary fistula	Days
1	Trotteur français, G, 5 years	R	E	No	‐	Yes	Yes	Yes	‐	‐	‐	4
2	Warmblood, M, 6 years	R	M	TACE	Yes	‐	‐	Yes	‐	Yes	‐	10
3	Thoroughbred, G, 13 years	L	M	TACE	Yes	‐	‐	Yes	Yes	‐	‐	10
4	No breed, S, 3 years	L	M	TACE	Yes	‐	Yes	Yes	‐	‐	‐	10
5	Spanish, G, 10 years	L	M	TACE	Yes	‐	‐	Yes	‐	‐	‐	10
6	Thoroughbred, G, 15 years	R	M	TACE	Yes	‐	‐	Yes	Yes	‐	‐	10
7	Warmblood, G, 19 years	R	M	TACE	Yes	‐	‐	Yes	‐	‐	‐	10
8	Warmblood, G, 4 years	R	M	No	Yes	‐	‐	Yes	‐	‐	Yes	10
9	Zangersheide, M, 4 years	L	M	No	Yes	‐	Yes	Yes		‐	‐	10
10	Trotteur français, G, 3 years	L	E and M	No	Yes	Yes	‐	Yes	‐	‐	‐	13
11	Thoroughbred, G, 8 years	L	M	TACE	Yes	‐	‐	No	Yes	‐	‐	10
12	Thoroughbred, G, 8 years	R	M	TACE	Yes	‐	‐	Yes	‐	‐	‐	10
13	No breed, G, 12 years	R	M	TACE	Yes	‐	‐	Yes	‐	‐	‐	10
14	Selle Français, G, 2 years	L	M	TACE	Yes	‐	‐	Yes	‐	‐	‐	17
15	Idem No. 14	R	M	TACE	Yes	‐	‐	No	‐	‐	‐	11

Abbreviations: E, empyema; G, gelding; L, left; M, mare; M, mycosis; P, procedure; R, right; S, stallion; TACE, transarterial coil embolization.

### 2.5 Surgical procedure

#### 2.5.1 Surgical team

The procedure required four people: one for sedation and equipment handling, one for endoscope manipulation, one for physical restraint and endoscopy assistance, and one for catheterization.

#### 2.5.2 Description of the catheter

The first six TGPC (Nos. 1–6) were performed using a 20 Fr × 25 cm silicone catheter (Percutaneous Balloon Catheter for Cystostomy, Kit PBC20, Mila International Inc., Florence, Kentucky). Based on these results, a more ergonomic catheter was developed to perform the next nine TGPC (Nos. 7–15). This 20 Fr diameter silicone, single‐lumen catheter (Equine Transcutaneous Guttural Pouch Balloon Catheter, Kit 9500‐BC, Mila International Inc) was characterized by a length of 10 cm from the distal open‐exit port to the hub, a 10 mL silicone retention balloon around the distal open‐exit, and a tethered cap on the hub side. Amovable fixation ring was added to provide 5 cm of space from the balloon when slid back to the catheter hub.

#### 2.5.3 Horse preparation

**FIGURE 2:**
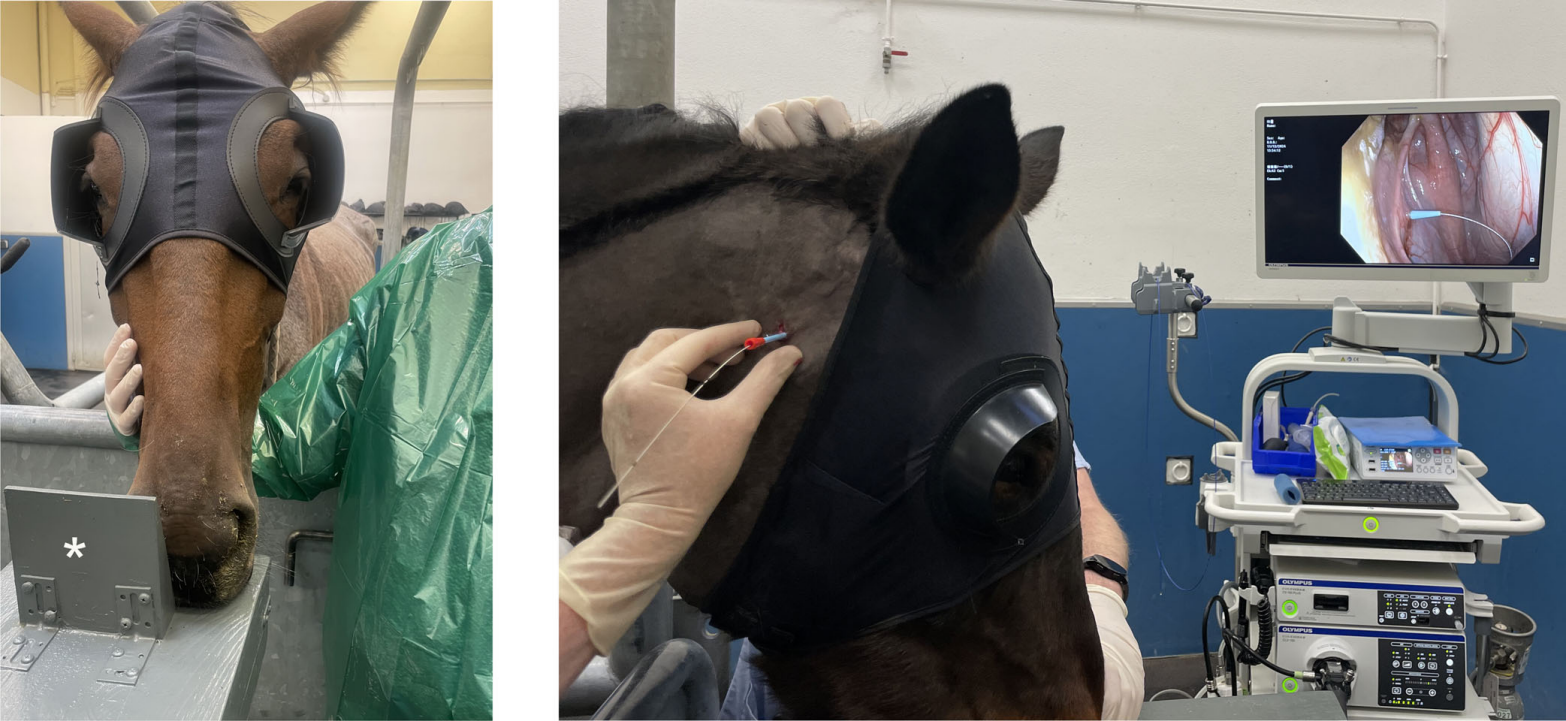
Photographs illustrating the setup for transcutaneous catheterization of a guttural pouch (GP). The horse blinder cap is placed, and the head is positioned at a 90° angle using a modified head support, ensuring that the nostril is clear on the side of the affected GP to allow passage of the endoscope (left). The endoscopy tower is placed on the opposite side of the GP to be catheterized to provide visualization for the surgeon (right). The endoscopic image displayed on the tower shows the 12 Fr tissue dilator passing around the stainless‐steel guidewire.

The horses were placed in a standing stock. A horse blind cap (Zilco International Pty Ltd., Sydney, Australia) that kept the parotid region clear was placed over the head (Figure 2). The mane was braided and a rope was placed around the top one‐third of the neck and through the braids to physically restrain the horse. The head was maintained at a 90° angle to the neck, with the chin placed on the head support and maintained in this position throughout the surgery using a rigid wedge attached to the head support (Figure 2, left). The endoscopy tower was placed on the opposite side of the planned GP procedure for ease of visualization (Figure 2, right). A metal probe (Endjow hot FD‐230 U, Olympus, Japan) was inserted in the working channel of the videoendoscope. On the side to be catheterized, an area from the base of the ear to the jugular groove and from the mandible to the wing of the atlas was clipped before being aseptically prepared. A 14 gauge 2.1 × 133 mm IV catheter (BD Angiocath, Becton Dickinson, Utah) was placed into the opposite jugular vein for sedation of the horse for the 20–30 min procedure. Sedation was obtained using detomidine, 20 μg/kg IV. If necessary, a half dose was administered again. Butorphanol, 0.025 mg/kg IV, was added to the protocol for the first two procedures. The patency of the catheter balloon was verified by injecting 10 mL of water prior to the procedure.

#### 2.5.4 Endoscopic landmarks

**FIGURE 3:**
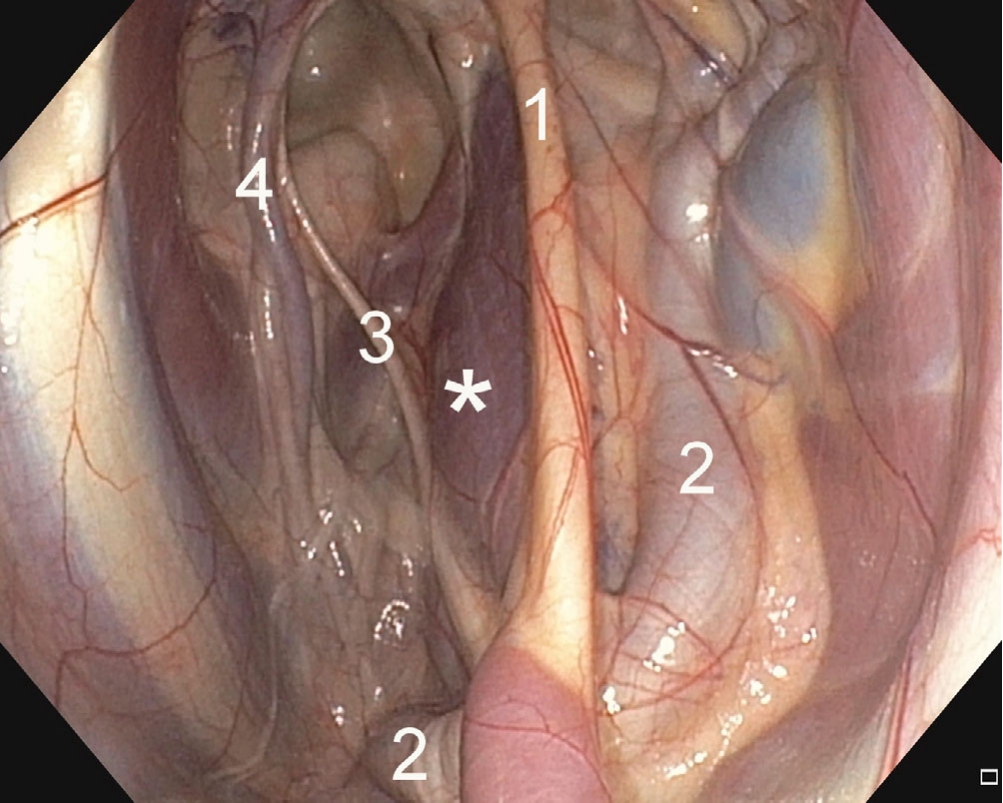
Endoscopic landmarks of the guttural pouch relevant to transcutaneous catheterization. Star = catheter penetration site into the stylohyoid muscle, 1 = stylohyoid bone, 2 = external carotid artery, 3 = CN XII (hypoglossal nerve), 4 = internal carotid artery.

For each horse (*n* = 14) that underwent TGPC, the endoscope was placed ventrally at the entrance of the medial compartment of the GP to be catheterized, and light pressure was applied to the skin with one finger at the CPS. The stylohyoid muscle should move to the desired position under the stylohyoid bone and at a certain distance from the ICA, ECA, and cranial nerves (Figure 3).

#### 2.5.5 Ultrasonographic examination

For TGPC 11–15, an ultrasound linear probe (Clarius HD3, Vancouver, Canada) was placed above the CPS to localize the maxillary vein.

#### 2.5.6 Catheterization procedure

**FIGURE 4:**
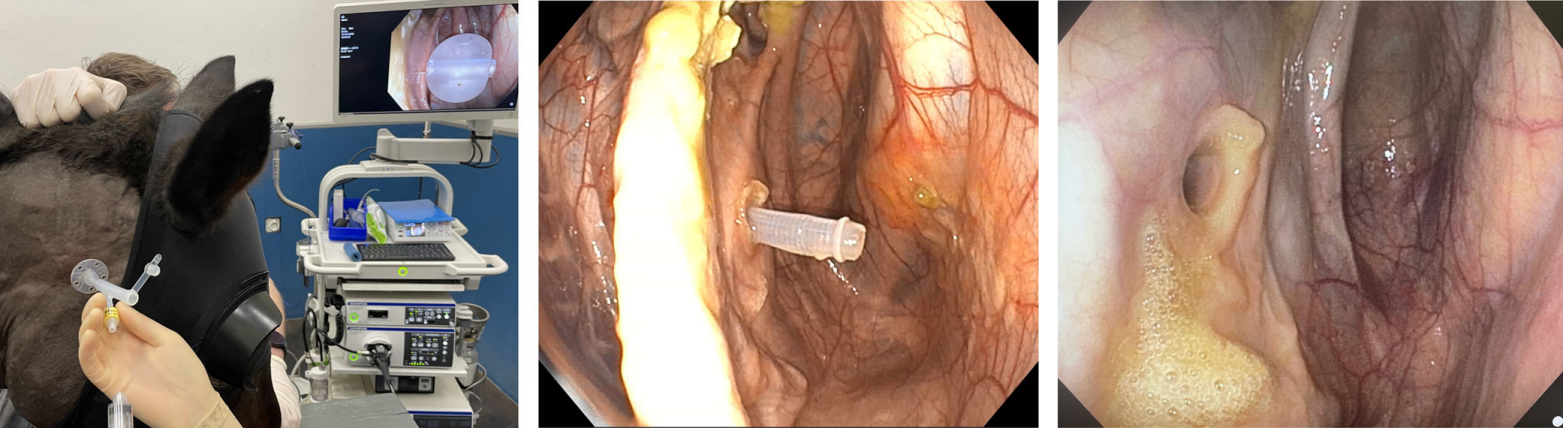
Photographs showing transcutaneous catheterization of a right guttural pouch, with the appearance of the balloon inflated (left), balloon catheter after removal of 10 mL of water for injection (center), and hole in the respiratory mucosa left immediately after balloon catheter removal (right).

The first step of TGPC before the surgical scrub was to locate the CPS (see 2.1.1, external body landmarks) and place a skin staple 1 cm below the CPS. The second step was to perform local anesthesia at the CPS using a 25 gauge needle, to inject 2 mL of 2% lidocaine subcutaneously. Using the metal probe placed in the working channel, the endoscope was advanced through the ipsilateral nasal cavity into the GP. After general inspection of the GP, the endoscope was positioned to obtain a good view of the entire area beneath the stylohyoid bone, particularly the stylohyoid muscle, in the medial compartment (Figure 3). Gentle pressure was applied to the skin at the CPS to endoscopically assess the correct catheter location by observing the back‐and‐forth movement of the stylohyoid muscle. A 16 gauge catheter‐over‐needle was inserted into the CPS perpendicular to the skin and subcutaneous tissue at a depth of 4 to 5 cm. Gradual detachment of the mucosa was observed before piercing the GP mucosal lining. Once the catheter was positioned in the medial compartment of the GP, the needle was removed. A 0.032″ × 45 cm stainless‐steel guidewire with a J‐tip was inserted into the catheter. Using a No. 11 scalpel blade the skin incision was extended to 2 cm. Leaving the guidewire in place, The catheter was removed and a 12 Fr tissue dilator was inserted. Firm pressure with a slight twisting motion was applied to dilate the tract until the dilator was seen in the GP and then advanced 1 cm (Figure 2, right). The horse handler applied equivalent counter pressure with one hand toward the base of the opposite ear to keep the head in line with the body. Maintaining the head at a 90° angle, and leaving the guidewire in place, the 12 Fr tissue dilator was removed and a 24 Fr dilator with a peel‐away introducer sheath was inserted. The guidewire was then grasped with one hand, and the 24 Fr dilator with a peel‐away sheath was advanced by twisting and pushing close to the skin. Equivalent counter pressure, performed by horse handler toward the base of the opposite ear, was needed at this stage. Once the 24 Fr peel‐away sheath was secured by at least 1 cm into the GP, the wire and the 24 Fr dilator were removed and replaced with the 20 Fr balloon catheter. The peel‐away sheath was slowly removed by grasping the blue handles with both hands and peeling them away from the hub to break them apart. The balloon catheter was advanced into the GP until the deflated balloon was clearly visible on endoscopy. The balloon was inflated with 10 mL of water for injection (Figure 4, left). The peel‐away sheath was then completely removed. The tethered cap was closed. The catheter was pulled slightly outwards to ensure that the balloon was adjacent to the mucosal lining of the GP, and the fixation ring was advanced to the skin level before being sutured with a 2–0 nylon suture on a straight needle. The fixation ring was left exposed or covered with a cotton pad included in the kit and fixed with an adherent bandage.

#### 2.5.7 Catheter removal procedure

The balloon catheter was removed under endoscopic visualization after light sedation (Figure 4, center and right). The contents of the emptied balloon were measured, and a sterile nonadherent dressing was applied for 3 days, after which the wound was left to heal by second intention.

## 3 RESULTS

### 3.1 Ex vivo anatomical study

**FIGURE 5:**
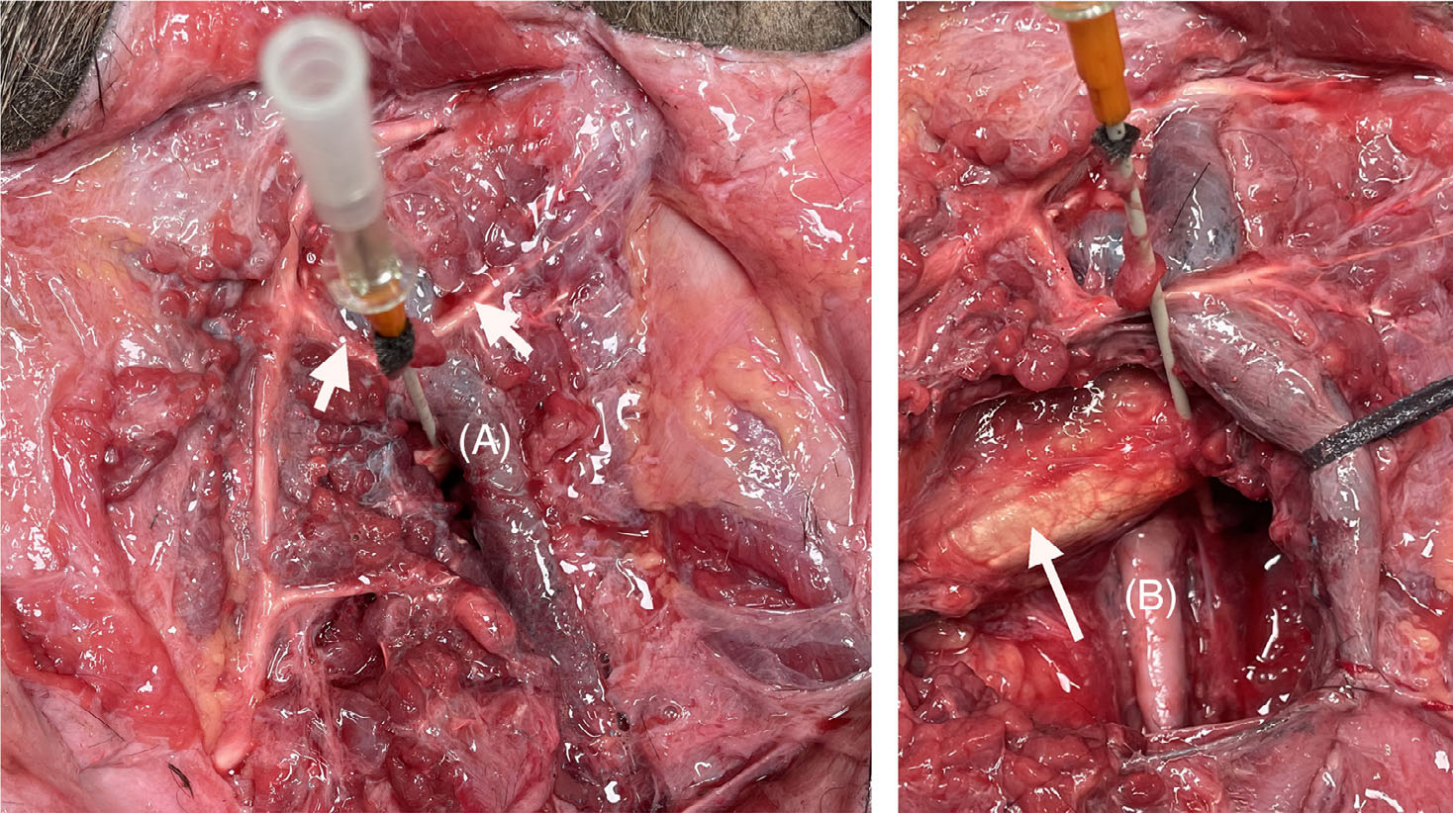
Photographs from a horse cadaver head demonstrating the anatomical structures around the catheter penetration site. Rostral is toward the left and caudal is toward the right. The catheter passes ventrally to the maxillary vein (A); three parotid duct tributaries to the main parotid duct are observed, with the middle one (arrow) located just dorsally to the catheter (left). The external carotid artery (B) is located deeper, above the lateral wall of the medial compartment. The catheter passes near the caudal edge of the digastric muscle, which is surrounded by a dense fibrous aponeurosis (arrow) (right).

This study showed that the catheter placed in the CPS passed through the skin, thin fascia, parotidoauricular muscle, parotid gland, stylohyoid muscle, and GP mucosa before entering the medial compartment of the GP. The skin and parotidoauricular muscles were reflected, and the parotid glands were dissected. In this cadaver specimen, the main duct of the parotid gland was located at the cranial border, parallel to the ascending branch of the mandible (Figure 5, left). Three secondary parotid ducts were visible: dorsal, middle, and distal. The catheter was positioned 1 cm away from the secondary middle parotid duct. The maxillary vein was located dorsal to the catheter. After removing the parotid gland around the catheter, the ECA (Figure 5, right) and hypoglossal nerve (CN XII) were located 1 cm ventral to the catheter. The catheter passed near the caudal edge of the occipitomandibular portion of the digastric muscle which was surrounded by a dense fibrous aponeurosis.

### 3.2 In vivo radiological study

The position of the staple representing the CPS in relation to the stylohyoid bone varied greatly among horses. The staple could be located in a space above the ventral edge of the stylohyoid angle and 3 cm further caudally; however, it was always superimposed on the GP.

### 3.3 History and clinical presentation

A total of 14 horses (11 geldings, 2 mares, and 1 stallion) with a median age of 7 years were included in the study (Table 1). A total of 10 horses with GP mycosis underwent a trans arterial coil embolization procedure (TACE) before or after TGPC. One horse had mycosis in both GPs without communication between them, requiring two TGPC procedures to treat both GPs simultaneously. With the exception of horses with severe dysphagia or respiratory infection on admission, horses did not receive antibiotics or anti‐inflammatory drugs for more than 3 days after TGPC, and no local treatment in the GP other than oxygen or 0.9% NaCl solution was administered.

### 3.4 Surgical procedure

The procedure required a team of four people. The same surgeon performed all the surgeries. The surgeon's proficiency improved as more surgeries were performed. Following inappropriate head movements during the first two surgeries, which were performed using a sedation protocol involving detomidine and butorphanol, butorphanol was omitted from the protocol for the next 12 surgeries. Positioning and stabilizing the head at a 90° angle using a modified head support was well tolerated by all horses. To protect the endoscope, the nostril on the catheterization side was kept clear from the rigid wedge (Figure 2, left). The horse blinder cap was well tolerated. The maxillary vein, approximately 1 cm below the skin, was easily identifiable on ultrasonographic examination, which helped adjust the CPS in procedures 11–15. When placing dilators, a certain amount of force with a slight twisting motion was required to pierce the different tissue layers. In procedures 1 and 8, it was necessary to apply greater pressure over a distance of approximately 1 cm when the dilators were advanced to a depth of 2–3 cm. This stage was facilitated if the assistant applied similar pressure on the opposite parotid region to keep the horse's head aligned with the body. The pressure required to advance the dilators increased with diameter.

In procedures 1 and 2, the fixation ring was fixed with four sutures at the 3, 6, 9, and 12 o'clock positions. Because the sutures at the 3 and 9 o'clock positions were no longer held after 10 days, only two sutures at the 6 and 12 o'clock positions were placed in the next procedures, except for one (No. 10). These sutures were still holding after 10 days, but abrasions were present under the fixation ring. These lesions resolved spontaneously within 5 days after removal of the balloon catheter without further treatment. In one procedure (No. 10), the ring was not sutured. A total of 10 days later, the catheter was still in place, but more significant abrasions had developed. These lesions persisted until the balloon catheter was removed on day 13. The wound then healed without further complications.

Once the balloon catheter was in place, it was possible to immediately begin administration of the planned treatments, that is twice daily lavage, with 1 L of 0.9% NaCl (No. 1 and No. 10) and administration of oxygen, four times daily, at a flow rate of 15 L/min (No. 2 to No. 15). In one horse (No. 10), the oxygen administration and lavage periods were at least 1 h apart. In two cases (No. 1 and No. 10), the balloon catheter was used at the end of the lavage session for passive drainage by leaving the balloon catheter open. In one case (No. 10), a 10 Fr silicone tube with a barbed adapter connected to suction was successfully inserted into the balloon catheter for active drainage to improve the evacuation of exudate remaining on the floor of the GP.

Endoscopy showed normal positioning and distension of the balloon at 4 days (No. 1), 5 and 10 days (all other cases), at 11 days (No. 15), 13 days (No. 10), and 17 days (No. 14). After removing the balloon catheter, the amount of water for injection collected (10 mL) from the balloon was the same as the volume instilled. Removal of the balloon catheter was performed without complications, leaving a slight defect in the mucosa (Figure 4, right) and a 5–6 mm hole in the skin. No tract infections were observed after catheter removal. Closure of the external wound was noted in all horses at 3 days, and all owners reported good healing 10 days after removal.

### 3.5 Intraoperative complications

There were only minor complications. In procedures 5 and 9, the 12 Fr dilator was correctly positioned; however, the 24 Fr dilator and its peel‐away sheath could not be advanced because of a bone structure. In procedure 5, after applying a skin staple at the CPS, a lateral radiograph of the head showed superimposition of the staple on the ventral edge of the stylohyoid bone. The CPS was moved from 3 cm to 3.5 cm caudal to the vertical ramus of the mandible, allowing the 24 Fr dilator and its peel‐away sheath to advance normally. In procedure 9, no radiographs were taken; however, moving the CPS 1 cm caudally allowed for proper insertion. Although the pressure required to advance the 12 Fr dilator was consistent, the 24 Fr dilator required more force in some cases.

Mild hemorrhage was endoscopically observed inside the GP in most procedures (13/15). This usually occurred when the 16 gauge catheter‐over‐needle introducer was removed or when the12 Fr dilator was inserted. In all cases, bleeding was temporary and consisted of a few drops. In three out of 15 procedures (No. 1, No. 4, and No. 9), venous bleeding of varying severity was observed at the skin level, either when the skin incision was enlarged with the No. 11 scalpel blade (No. 1) or when the horse started to wake up and move its head, even though the balloon catheter, covered by its dressing, had already been in place for a few minutes (No. 4 and No. 9). In these three cases, a compression head bandage consisting of two gauze rolls, placed in the parotid region on either side of the balloon catheter and covered with an adhesive bandage around the head, was applied. In one procedure (No. 4), venous blood still passed through the compression head bandage, which required the addition of an extra layer of bandage. The compression head bandage was removed after 12–24 h, with no complications or bleeding recurrence. In one procedure (No. 9), upon removal of the head bandage, a clot between the skin and the fixation ring was removed using a hemostat.

### 3.6 Postoperative complications

Only minor complications were reported following surgery. During the treatment of one horse (No. 2), oxygen was mistakenly administered through the balloon inflation tube, causing it to rupture in the GP. During oxygen administration, sudden backward movements caused the inflated balloon catheter to exit the GP while still attached to the oxygen system in three horses (Nos. 3, 6, and 11). These reactions were unrelated to the procedure itself. The balloon was emptied, and the catheter was successfully repositioned within 6 h using a rigid stylet and endoscopic guidance. In one horse (No. 8), the dressing placed after removal of the balloon catheter fell off after 24 h. To apply the new dressing, the horse was fed treats to avoid sedation. The attraction of food and chewing induced salivation, causing a few drops of saliva to flow through the CPS. This flow was reduced at 48 h and was absent at 72 h, allowing the horse to be discharged. No other parotid sequelae were reported during treatment or during the 10 days period following the removal of the balloon catheter.

### 3.7 Outcomes

No patient showed infection at the CPS, and second‐intention healing was achieved within 10 days after catheter removal in all cases. No dressing or special care was required on the third day after removal. More than 500 sessions of topical oxygen therapy (30–45 min each) were performed without sedation in 13 horses. In two individuals (Nos. 1 and 10), GP lavage was performed using 1 L of 0.9% NaCl solution injected manually via the balloon catheter. A small amount of exudate was evident through the ipsilateral nostril. No cases of neuropraxia related to the procedure were reported, even when both GPs were catheterized (Nos. 14 and 15) for more than 2 weeks.

## 4 DISCUSSION

Despite the complex anatomy of the GP, this study shows that a transcutaneous approach to the GP through the parotid region using a 20 Fr balloon catheter provides access for administering treatments within the GP for several days. This is followed by uncomplicated surgical site healing in less than 10 days after the catheter is removed, with no further care required after 3 days. TGPC is performed standing and can be conducted outside the surgical theater when dealing with a contagious *streptococcal* infection.^7^ The technique is also advantageous when dysphagic horse is fed through a nasoesophageal tube, where a transnasal approach to place a GP catheter is not an option. When a TACE procedure was performed before or after TGPC, the surgical site was at least 70 cm away from the CPS and did not cause any interference. When endoscopic visibility in the GP is impaired, it is advisable to perform the TACE procedure first to prevent the consequences of possible trauma to the ICA or the ECA. Following the observation of undesirable head movements during the first two procedures, butorphanol use was discontinued, as its combined use with detomidine can induce head jerking.^12^

Localization of the CPS using external endoscopic landmarks is essential. Palpation of the mandibular ramus is sometimes complicated by the presence of soft tissue. Before scrubbing, it is advisable to place a permanent marker, such as a skin staple, 1 cm underneath the CPS so that it can be easily located should the horse move. Correct placement of the 16 gauge catheter‐over‐needle introducer at the CPS, perpendicular to the skin, is essential since successive dilators only push the tissues back, reducing the risk of major vessel or cranial nerve trauma. During advancement of the dilators, the surgeon may experience significant resistance, which may be because of the stylohyoid bone or the dense fibrous aponeurosis of the digastric muscle. If the surgeon feels that the end of the dilator is slipping onto the bone, it is recommended to completely exit and modify the CPS to increase the distance from the vertical ramus of the mandible by 0.5–1 cm. A radiological study explains this phenomenon by the variations in size and shape of the stylohyoid angle where three muscles attach.^13^ If no bony sensation is felt, increased resistance can be attributed to the passage of the dilators through the digastricus muscle.

The balloon catheter used in procedures 7–15 can be placed through a thickened parotid area of up to 50 mm. The integrated cap on the hub side can be left open for passive drainage or to change the GP environment.^14^ After insertion, the retention balloon needs to be immediately inflated to prevent the catheter from exiting the GP because of inappropriate movement. A total of 10 mL of water for injection was optimal to distend the balloon, maintaining sufficient space to allow treatment administration without obstruction by the ventromedial mucosal lining of the GP. Under normal conditions, the balloon was solid yet deformable. On three occasions during treatment, it emerged intact after the horse moved backward quickly. All catheterization sites showed varying degrees of skin abrasion under the fixation ring. The least reactions were observed when the fixation ring was fixed using two sutures placed in an axis parallel to the vertical ramus of the mandible. The largest lesion was observed when the fixation ring was not sutured, which can be explained by greater friction during head movements. Penetration of the stylohyoideus muscle in all cases of TGPC and probable unintentional penetration of the digastric muscle in two procedures did not lead to any change in the horse's health, such as the development of dysphagia.

Although the transnasal technique for placing catheters in a GP has been described,^15^ the volumes of fluid or oxygen that can be delivered are limited by the diameter (2.3 to 2.6 mm) of these catheters, and they can be source of discomfort in the nostril if an indwelling catheter is used. The largest‐diameter balloon catheter used in this study (6.7 mm in diameter) was effective in all cases when used to repeatedly deliver 15 L/min of oxygen or 1 L of lavage solution to fill the GP. The fact that the pharyngeal ostium was free of a catheter or an endoscope enabled evacuation of excess gas or liquid, reducing the risk of inducing rupture of the GP.^1^

Endoscopically, a few drops of blood were typically visible at the mucosal level when the 16 gauge introducer was removed. This phenomenon is probably caused by blood from the tissue layers penetrated by the introducer. More importantly, venous bleeding, which was observed during three procedures, was likely because of maxillary vein trauma. Bleeding stopped in all cases after application of a compression bandage. This complication was of no clinical significance during the catheterization period or the subsequent 10‐day follow‐up. However, transverse ultrasonographic examination of the CPS, performed in five TGPCs (Nos. 11–15) to locate the maxillary vein prior to sterile scrubbing, was effective in preventing such bleeding.

The observation of a few drops of saliva after balloon catheter removal in one horse (No. 8) suggested that the secondary middle parotid duct may have been damaged during the procedure and it is possible that other small lesions of the parotid gland were not observed on clinical examination during other procedures. Saliva contains mucins, enzymes, and immunoglobulins that contribute to its lubricating and antimicrobial properties.^16^, ^17^ This may explain the absence of infection at the CPS in this study. Administration of a lavage solution^15^ or oxygen^18^ through the balloon catheter may also be beneficial in preventing infection.

The minimally invasive nature of TGPC is evident from the fact that it was not associated with any major complications or neurologic deficits, and that all horses maintained excellent mobility of the head and neck, even when a balloon catheter was placed in each GP (No. 14 and No. 15) in the same horse. However, endoscopic guidance is essential to ensure that vessels and nerves are not damaged.^2^ Catheter dislodgement due to unexpected head movements was a rare complication during topical oxygen therapy sessions,^3^ but the catheter can be easily replaced within 24 h, before the hole contracts and a fibrin plug is deposited at the CPS. This complication can be prevented by ensuring a detachable connection between the balloon catheter and oxygen administration system.

Based on our results, we can conclude that all adult horses with bacterial or fungal infections of the GP included in the study could be treated safely as often and for as long as desired. The transcutaneous insertion of a 20 Fr balloon catheter placed into the medial GP compartment via a minimally invasive transcutaneous approach can be considered a valid surgical option to administer a local treatment. Adherence to body and endoscopic landmarks during balloon catheter placement is essential. The most common minor complications are trauma to the maxillary vein and skin abrasions under the fixation ring. The former can be prevented using preoperative ultrasound, while the latter heals naturally within a few days. The advantages of TGPC over existing techniques include: an external approach in sedated horses, the possibility of repeating treatments in one or two GPs simultaneously, without causing the horse discomfort, rapid wound closure and the ability to perform the procedure on the field, without the need for a laser.

## AUTHOR CONTRIBUTIONS

Lepage H: Study organization, data collection and analysis, drawing, and manuscript preparation. de Chaisemartin C, DMV: Data collection, analysis, and manuscript preparation. Spadaro Rosselo A, DVM: Data collection. Leroy H, DMV, PhD: Data collection. Lepage O, DMV, PhD, DECVS: Study design and organization, surgeon, data analysis, and manuscript preparation. All the authors approved the final version of the manuscript.

## FUNDING INFORMATION

No grant or external funding was used for this study.

## CONFLICT OF INTEREST STATEMENT

The authors declare no conflicts of interest.

## Data Availability

Absence of shared data.
